# Tools and recommendations for commissioning and quality assurance of deformable image registration in radiotherapy

**DOI:** 10.1016/j.phro.2024.100647

**Published:** 2024-09-14

**Authors:** Lando S. Bosma, Mohammad Hussein, Michael G. Jameson, Soban Asghar, Kristy K. Brock, Jamie R. McClelland, Sara Poeta, Johnson Yuen, Cornel Zachiu, Adam U. Yeo, the 2021 ESTRO Physics Workshop on Commissioning and Quality Assurance for Deformable Image Registration in Radiotherapy

**Affiliations:** a Department of Radiotherapy, University Medical Center Utrecht, Utrecht, the Netherlands; b Metrology for Medical Physics Centre, National Physical Laboratory, Teddington, UK; c GenesisCare, Sydney, Australia; d School of Clinical Medicine, Medicine and Health, University of New South Wales, Sydney, Australia; e Oncology Systems Limited, Shrewsbury, UK; f Department of Imaging Physics, The University of Texas MD Anderson Cancer Center, Houston, TX, USA; g Centre for Medical Image Computing and the Wellcome/EPSRC Centre for Interventional and Surgical Sciences, Dept. Medical Physics and Biomedical Engineering, University College London, London, UK; h Medical Physics Department, Institut Jules Bordet – Université Libre de Bruxelles, Belgium; i St. George Hospital Cancer Care Centre, Sydney NSW2217, Australia; j Ingham Institute for Applied Medical Research, Sydney, Australia; k Peter MacCallum Cancer Centre, Melbourne, VIC, Australia; l The Sir Peter MacCallum Department of Oncology, the University of Melbourne, Melbourne, VIC, Australia

**Keywords:** Quality Assurance, Commissioning, Validation, Verification, Deformable image registration, Dose warping

## Abstract

Multiple tools are available for commissioning and quality assurance of deformable image registration (DIR), each with their own advantages and disadvantages in the context of radiotherapy. The selection of appropriate tools should depend on the DIR application with its corresponding available input, desired output, and time requirement. Discussions were hosted by the ESTRO Physics Workshop 2021 on Commissioning and Quality Assurance for DIR in Radiotherapy. A consensus was reached on what requirements are needed for commissioning and quality assurance for different applications, and what combination of tools is associated with this.

For commissioning, we recommend the target registration error of manually annotated anatomical landmarks or the distance-to-agreement of manually delineated contours to evaluate alignment. These should be supplemented by the distance to discordance and/or biomechanical criteria to evaluate consistency and plausibility. Digital phantoms can be useful to evaluate DIR for dose accumulation but are currently only available for a limited range of anatomies, image modalities and types of deformations.

For quality assurance of DIR for contour propagation, we recommend at least a visual inspection of the registered image and contour. For quality assurance of DIR for warping quantitative information such as dose, Hounsfield units or positron emission tomography-data, we recommend visual inspection of the registered image together with image similarity to evaluate alignment, supplemented by an inspection of the Jacobian determinant or bending energy to evaluate plausibility, and by the dose (gradient) to evaluate relevance. We acknowledge that some of these metrics are still missing in currently available commercial solutions.

## 1 Introduction

Registration and fusion of medical images has become an integral component of a wide range of procedures within radiation oncology which are increasingly being used to inform and drive clinical decisions [1], [2], [3]. Target and/or organ-at-risk delineation, image-guided treatment, response assessment, re-planning and plan adaptation are example procedures in a patient’s treatment workflow which are now generally underpinned by image registration and fusion processes. These processes typically manipulate multimodal, anatomical atlas and/or time-series image data and their use in radiotherapy is expected to increase in the near future [3], [4], [5], [6], [7], [8]. However, it should be recognised that image registration is a complex process and spatial registration uncertainties are routinely observed after the image registration or fusion has been performed. In this context, guidance to assist the commissioning, quality assurance (QA), and clinical integration of image registration and fusion techniques is necessary before its routine use in clinics. The scarcity of (adequate and easy to use) tools for commissioning and QA was identified as a key barrier to the clinical use of deformable image registration (DIR) in recent international surveys [3], [4].

**Fig. 1:**
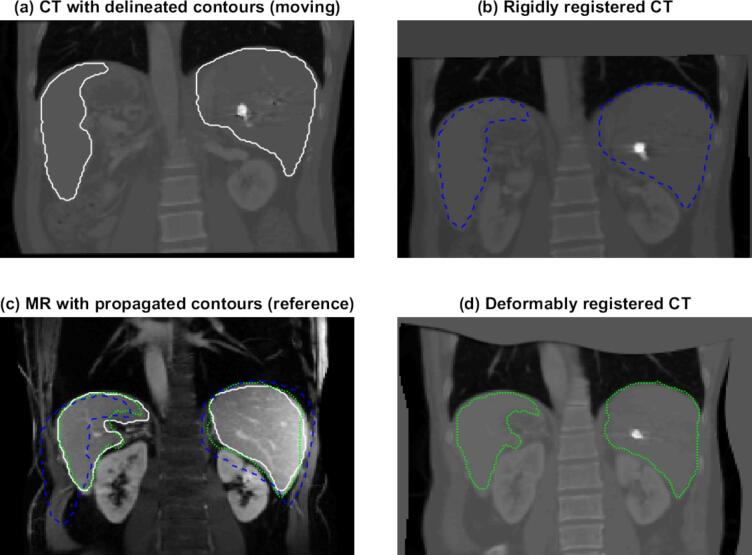
Example of using image registration for contour propagation from a (moving) CT image to a (reference) MR image of the abdomen. Both the result from rigid (blue dashed line, panel (b)) and deformable (green dotted line, panel (d)) image registration are shown. Panel (c) shows these same propagated contours on the MR image, together with the reference contours in white. Images and delineations are adapted from Hering et al. [13]. (For interpretation of the references to colour in this figure legend, the reader is referred to the web version of this article.)

**Fig. 2:**
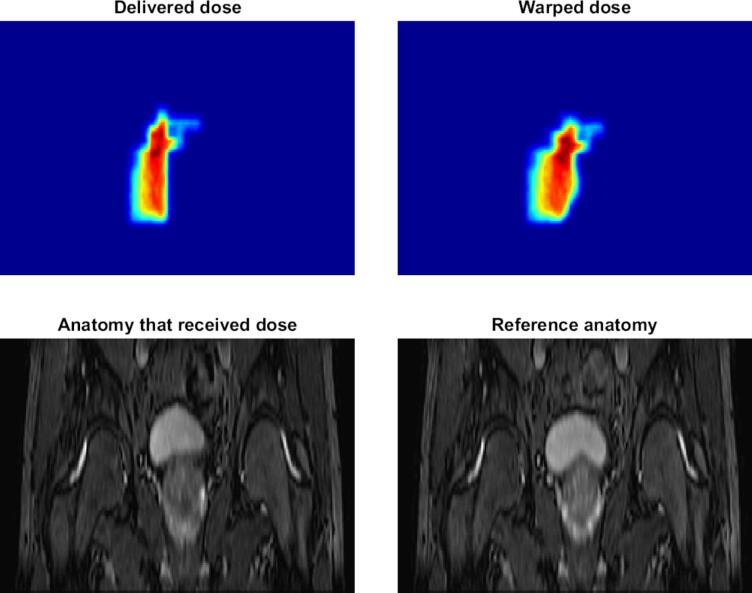
Example of applying deformable image registration to warp a (partial) dose distribution for reconstruction of intra-fraction delivered dose for a prostate cancer patient on a MR-linac.

The report of the American Association of Physicists in Medicine (AAPM) Radiation Therapy Committee Task Group No. 132 [9] published in 2017 reviewed rigid image registration and DIR solutions and provided recommendations for commissioning and QA of clinical image registration and fusion techniques in radiotherapy. The report mentions that DIR for dose accumulation is outside its scope. When evaluating image registration for contour propagation (see Fig. 1), the aim is generally unambiguous. If the propagated contours overlap sufficiently well with the organs or an operator’s delineations, the registration solution is deemed reliable. However, when the application of DIR is warping quantitative information (such as radiation dose, Hounsfield units, PET intensity, see Fig. 2) where each voxel holds information, assessing the validity of a registration process requires a more comprehensive approach. For dose warping, in particular, there is a nontrivial relation between registration errors and dose warping errors, which should be reflected in the evaluation [10], [11], [12].

Discussions were held during the ESTRO Physics Workshop 2021 on “commissioning and quality assurance of DIR for current and future RT applications”. The workshop included focused discussions on best practices and considerations for dose mapping/accumulation, presented in [14], on recommendations for DIR uncertainties, presented in [15], and on recommendations for commissioning and QA tools, presented in this article. Online meetings and discussions were held between May 2021 and September 2023. We made a comprehensive overview of available metrics and tools and defined inputs, outputs, and limitations. A consensus was then reached on what (complementary) requirements are needed for commissioning and QA of DIR for applications like contour-propagation or dose accumulation, and what tools and metrics are recommended for these different applications, based on this. The group endorses the recommendations of the report of AAPM task group 132 [9] and expands on the report by addressing additional tools and providing reasoning for using a set of tools, depending on the requirements for the intended application. We assess how well the tools presented in the AAPM report still hold-up given the current status of adaptive radiotherapy. In particular, we expand on application-specific recommendations for evaluation of DIR for warping quantitative information, especially for dose accumulation. We believe that improved and standardised QA and commissioning will aid the safe clinical adoption of DIR. Being able to reliably assess the error or uncertainty in the registration outcome will help to trust this result. Additionally, standardised assessment of the registration outcome allows a comparison to the result without registration (or after rigid registration), indicating potential benefits of using DIR.

## 2 Tools for quality assurance and commissioning of DIR

**Fig. 3:**
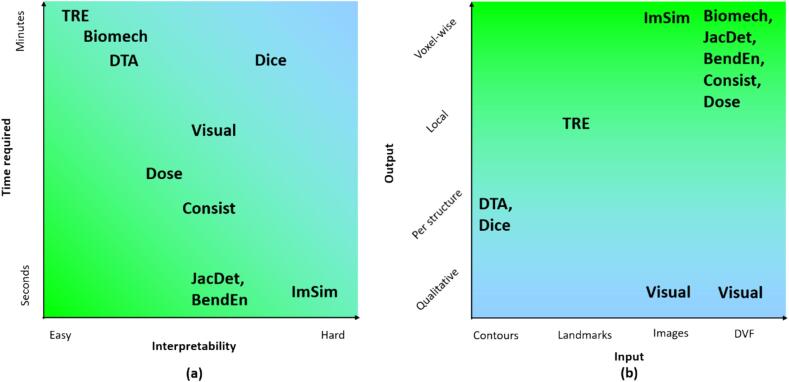
(a) Overview of the ease of interpretation and time required for different metrics. Fast and easy to interpret metrics are useful for (semi-automatic) patient-specific and registration-specific quality assurance. (b) Overview of the input and output of different metrics. The voxel-wise outputs in the top row are especially useful for evaluating DIR for dose warping/accumulation. The metrics are image similarity (ImSim), biomechanical criteria (Biomech), the Jacobian determinant (JacDet), bending energy (BendEn), consistency metrics (Consist), dose-based metrics (Dose), the target registration error (TRE), the distance to agreement (DTA), the Dice similarity metric (Dice), and visual comparisons (Visual).

Many tools and metrics are available for QA. Generally, individual tools and metrics are necessary but not sufficient [16]. Therefore, a combination of complementary tools is needed to achieve or approach sufficiency. An overview of selected tools and their characteristics is given in Table 1. These characteristics are the input used, the output given, the time required for evaluation, general acceptance limits when using these metrics, and considerations involved in using these metrics. In Fig. 3, selected metrics are organized based on their timing and interpretability, and on their input and output. Below we expand on this and describe available tools for commissioning and QA. The metrics are sorted according to what they evaluate and how they perform the evaluation.

**Table 1:** Overview of various tools for commissioning and quality assurance and their characteristics: the input required, the kind of output generated, their general acceptance limits, their evaluation time, and (dis-)advantages or considerations for choosing a set of tools to use. DVF indicates the deformation vector field.

Tool/metric	Input	Output	Timing	General limits	Considerations
Visual inspection	Images	Qualitative assessment	Minutes		Include expertise, interpretation, not quantifiable, hard to see some errors
Dice similarity coefficient	Contours	Per structure	(Tens of) minutes^1^	0.75–0.90	Need contours, unclear interpretation, depends on shape and volume, historical interpretation
Mean, max or percentile distance to agreement (DTA)	Contours	Per structure, distributional, mm	(Tens of) minutes^1^	2–3 mm	Need contours, local description
Target registration error (TRE)	Landmarks	Local, mm	Tens of minutes^2^	2–3 mm	Need landmarks, local description
Normalized mutual information (MI)	Images	Voxel (using a window)	Seconds		Careful when assessing DIR using MI, hard to interpret
Structural similarity (SSIM)	Images	Voxel (using a window)	Seconds	0.8–0.9	Careful when assessing DIR using SSIM, only mono-modal, hard to interpret
Normalized cross correlation (NCC)	Images	Voxel (using a window)	Seconds	0.8–0.9	Careful when assessing DIR using NCC, only mono-modal, hard to interpret
Modality independent neighbourhood descriptor (MIND)	Images	Voxel (using a window)	Seconds		Careful when assessing DIR using MIND, hard to interpret
Inverse consistency	DVF	Voxel	Tens of seconds	Twice the voxel resolution	Requires additional inverse registration
Transitivity error	DVF	Voxel	Tens of seconds	Twice the voxel resolution	Requires multiple images and registrations. Gives algorithm uncertainty instead of registration uncertainty.
Distance to discordance	DVF	Voxel	Tens of seconds	0.4 for 2 mm	Requires at least 4 images. Requires (n-1)! registrations for n images as well as inverse transformations. Gives algorithm uncertainty instead of registration uncertainty.
Jacobian determinant (JacDet)	DVF	Voxel	Seconds	Close to 1 except for expanding/compressing or (dis)appearing tissue and sliding interface; [0, 2]; specific ranges for bones, liver, kidney	
Curl magnitude	DVF	Voxel	Seconds	Between 0 and 1; specific ranges for bones, liver, kidney	
Bending energy (BendEn)	DVF	Voxel	Seconds		Inversely proportional to smoothness
Tensile & shear mechanical stress	DVF, Poisson ratio + elastic modulus, shear modulus	Voxel	Seconds	Tissue-specific physiological limits	Need contours and tissue characteristics
Compare doses on different anatomies	CT’s, dose distribution, dose engine, DVF, (contours)	Voxel, Gy, (DVH)	Tens of minutes	Within 5 %	Requires dose recalculation
Dose error as combination of registration error and dose distribution	Images, dose distribution	Voxel, Gy	Minutes	Within 5 %	Requires a voxel-by-voxel registration error estimation

^1^For generating the contours.

^2^For generating the landmarks.

### 2.1 Visual

Visual assessment based on the experience of an operator is the important first step of any QA or commissioning procedure. Depending on the preference of the operator, the registration result can be visualized using e.g. image overlay, checkerboards, image differences, or image fusion. If the application is contour propagation, the warped contours can be shown on the image. For applications where the full deformation vector field is of interest, this can be visualized using e.g. a heat map, deformation grid, or by showing the vectors over the image. Additionally, the average (mean, max) of the DVF magnitude or a DVF-magnitude-volume histogram can be shown to indicate the extend of the deformations. Visual inspection gives a broad indication of the quality of a registration. The disadvantage is the lack of quantification which results in an unclear interpretation. Visual inspection is a necessary element of any evaluation. For QA of contour propagation or rigid registration, visual inspection may be sufficient.

### 2.2 Contours & landmarks

To quantify the registration performance using operator expertise, contour or landmark correspondence can be used. The benefit of these methods is their use of manual expertise, providing an independent validation. In fact, a manual evaluation is the only test that is guaranteed to be unbiased for any (future) registration algorithms.

A disadvantage for all operator-based methods is: (a) the inherent uncertainty in manually identifying anatomical boundaries and landmarks (introducing intra- and inter-observer variability [17]), (b) these are only identifiable in contrast-rich areas, (c) the selection process takes time, and (d) the evaluation is done locally only where contours or landmarks are available.

Contour correspondence can be quantified using volume overlap (Dice similarity coefficient, Jaccard index, concordance index), the centre of mass distance, or some boundary distance. For the boundary distance, many different methodologies and terminologies exist. We recommend using the terminology *mean/percentile/maximum distance to agreement* or, when communicating beyond the radiotherapy community, the *mean/percentile/maximum surface distance.* For the maximum distance to agreement, the historically used term Hausdorff distance can be used. Note that substantial differences were observed between implementations [18]. Using a percentile (often 90, 95, or 99) of the distance distribution can provide a balance between sensitivity and robustness. When comparing two contours, a distribution of the surface distance provides most information.

Distance measures are preferred over volume overlap criteria as they are more straightforward to interpret, can provide a distribution over contour points, and are independent of contour volume and shape [19], [20]. The Dice similarity coefficient can be used for comparing registration results on the same images and structures, when taking its limitations into account.

Contour correspondence may be sufficient for QA of contour propagation, but relates to registration performance only at the surface of delineated structures. Additionally, these metrics are unsuitable for online QA as they require volumes to be delineated. An in-depth overview of metrics for QA for (automatic) segmentation is given by Maier-Hein *et al.* [21].

The target registration error of a set of manually annotated anatomical landmarks is the benchmark and a necessary ingredient for the commissioning of DIR. It makes use of independent operator expertise and is straightforward to interpret. For patient-specific QA, the manual process of selecting these landmarks is time-intensive. Manually selecting 100 landmark pairs takes about 40–60 min [22]. A semi-automatic framework has been introduced, reducing the annotation time for 100 landmark pairs to 20–30 min [23]. Automatic frameworks for landmark pair generation have been introduced using vessel bifurcations [24], scale invariant feature transform (SIFT) points [22], [25], [26] and speeded-up robust features (SURF) points [27]. These methods reduce the annotation time to a few minutes and may help to create more landmark-datasets to be used for commissioning.

### 2.3 Image similarity

Many DIR algorithms use image similarity metrics as part of the objective function that the registration tries to optimise. When chosen carefully, some image similarity tools can also be used to assess the registration error. Previously suggested metrics are the cross-correlation [28], the mean squared error, mutual information [29] structural similarity [30] and the modality independent neighbourhood descriptor (MIND) [31]. These criteria can also output a value per voxel, either directly or when calculated using a small window around each voxel. Thereby, they can provide a distribution of errors or an error map [32]. Using similarity measures to evaluate a registration that includes those terms in its cost function will give biased results, especially when compared to algorithms that do not use these terms. Therefore, these metrics should be used with caution. However, they can provide useful information. Additionally, low image similarity can be used to indicate areas that require closer examination. A disadvantage of these metrics is that their output is not straightforward to interpret and it is difficult to put limits on what acceptable values are, as these heavily depend on the specific images and their modalities. Their advantages are their speed and that they provide a value per voxel.

When warping quantitative information, we recommend using at least one image similarity map to guide visual inspections. We also recommend evaluating the image similarity of the area of interest before and after registration for QA of DIR for warping quantitative information, especially for (semi-)automatic patient-specific QA.

Recently, neural networks have been introduced that estimate the voxel-by-voxel registration error [33]. Approaches include modelling a nonlinear relationship between the similarity measure and registration error trained on patient-specific model-generated data [34], estimating the registration error from image similarity trained on synthetic deformations [35], and trained on manually annotated landmark images [36], [37]. An in-depth overview is given by Bierbrier *et al.* [38]. This approach is promising, as these methods can potentially deliver voxel-by-voxel registration error maps. Further research is required as some of the usual obstacles of neural networks like sensitivity to unseen test cases, black-box behaviour, and potential for underestimating errors are especially important in the context of commissioning and QA.

### 2.4 Transformation consistency

Different assumptions on the nature of transformations can be used to assess the registration result. While consistency does not promise accuracy, inconsistency can indicate a lack of precision. When transforming image A to B and then image B to A, one might expect the combined displacement to be zero. Deviations from this are captured in the inverse consistency error [39], [40].

Similarly, a transformation for A to B to C to A is expected to be zero. The absolute magnitude of this transformation is the transitivity error [41], [42]. The distance to discordance metric is an extension of the transitivity error to at least four images [43]. A disadvantage of these two methods is that at least three and four images are needed. And as a consequence, the estimated error gives information on the performance of the algorithm on a dataset, not of a particular registration result. Although the output is in millimetres, it was previously found that the distance to discordance metric underestimates the registration error by about a factor of 4 [44].

### 2.5 Registration consistency

A similar argument can be used to test for a DIR-specific bias. Using multiple algorithms with different underlying assumptions on the same registration case can give an indication of the impact of these assumptions on the result. If the algorithms give similar results, this shows that there is no method-specific bias based on one of the assumptions. How exactly to interpret (considerable) differences is, on the other hand, not so straightforward. In some cases, it is also informative to evaluate the result of varying algorithm parameters, although the interpretation of the resulting variation is not straightforward and this should be done with caution.

### 2.6 Physical integrity of the transformation

Registration algorithms attempt to align images in a way that is useful and/or meaningful. This is not necessarily a representation of the underlying true physical process that has occurred between the images. Still, assumptions on these processes can be used to assess the plausibility of the registration result.

Generally, deformations are expected to be spatially smooth, meaning that nearby voxels move to nearby voxels. The degree of smoothness can be assessed using the bending energy [45], harmonic energy (the norm of the transformation [40], [46]) or the Dirichlet energy [47]. An advantage of these metrics is that they provide a value per voxel and are fast to compute. Their disadvantage is that there is no straightforward interpretation for their outcome values.

The Jacobian determinant of a deformation vector field provides information on the estimated volume change and its invertibility [48]. A Jacobian determinant of 1 indicates no volume change, while smaller and larger values correspond to a decrease and increase of volume. For incompressible regions like bones or watery tissues, the Jacobian determinants are thus expected to be close to 1. Negative Jacobians indicate that the transformation is noninvertible and implies tissue folding or sliding motion. Values above 2 indicate extreme expansion and may also be indicative of physically implausible deformations. In these cases, one should be aware of the effect of such a transformation on warping quantitative information. It is thus instructive to examine the number/percentage and the location of voxels with Jacobian determinants below 0 and above 2. For patient-specific QA we recommend inspecting the harmonic energy and/or Jacobian determinant.

From tagged MR images, it was found that the Jacobian determinant should range between [0.85, 1.10] for livers and between [0.94, 1.07] for kidneys [49]. Experimentally establishing Jacobian determinant ranges based on tagged MR images for more anatomies would be useful for QA and commissioning of DIR.

The curl magnitude of a deformation vector field gives the magnitude of vortices [50]. Generally, large curl values inside homogeneous tissue regions are not expected. The experimentally estimated values for intra-fraction motion are below 0.2 for livers, and below 0.1 for kidneys [49].

Biomechanical criteria can be used to determine whether the deformation vector fields abide the tissue biomechanical properties. Physical quantities such as tensile and shear mechanical stress were recently proposed for QA of DIR [49]. The tensile stress can be compared to tissue-specific thresholds for plastic deformation and the arterial blood pressure. The shear mechanical stress can be compared to thresholds for tissue fracture or rupture. These criteria can thus indicate if and where a registration estimates physiologically implausible deformations. Moreover, they have more direct biomechanical interpretations than the harmonic energy and Jacobian determinant, but do require segmentations of specific organs as well as their tissue characteristics. If these are available, we recommend using these metrics in the commissioning process.

### 2.7 Warping quantitative information

For applications involving the warping of quantitative information, specific methods for QA and commissioning have been introduced.

DIR can be used to deform the Hounsfield units from a planning CT to a daily cone-beam CT or MR image in order to perform a dose calculation on the anatomy of the day. For this application, the goal of the evaluation is clear as the resulting dose distribution should mimic the dose distribution calculated if a new CT was acquired for the anatomy of the day. Indeed, this has been used to evaluate the impact of registration errors for this application by using a second planning CT (aligned with the CBCT or MR) as the benchmark [51].

For dose warping applications, when corresponding anatomical landmarks are available in each image, the dose to the landmark can be sampled from both the original and mapped dose distributions. With accurate registrations, these doses should be equal. Clearly, this only provides a local assessment. The advantage is that this manual procedure is guaranteed to be unbiased for any future algorithms. Similarly, when delineated structures are available for both anatomies, these can be used to compare dose volume histograms and their parameters. This is particularly useful for CT-to-CT registrations, where doses can be recalculated [52]. Without structures, one can assess dose differences or the gamma index [53]. A disadvantage is that these methods take time, as dose delivery calculations have to be performed and landmarks or contours need to be created. This method shows promise for commissioning as well as e.g. QA for retrospective dose reconstruction.

To visualize the potential impact of the registration uncertainty on the warped dose, the distance to dose difference was introduced [54]. Given a dose distribution, it indicates how large a registration error can be before it introduces a predetermined maximum dose mapping error.

The magnitude of dose uncertainty was introduced as the maximum minus the minimum of the doses within a sphere around a voxel, where the radius of the sphere is determined by a predictor of the registration error [55]. The method provides a straightforward computation to find the maximum possible effect of a predicted registration uncertainty on the dose warping error for a certain spatial dose distribution. As the direction of a (predicted) registration error is not known, considering this *worst case* makes sense. This does, however, decrease the ease of interpretation of this metric. Depending on the preference of the operator, any visual inspection for QA of applications involving dose warping should include a map of the dose, dose gradient, the distance to dose difference, or the magnitude of dose uncertainty next to registration error predictors and the deformation vector field.

Finally, a few methods for estimating a voxel-by-voxel dose warping error were proposed that include the directional information of a predicted registration error. These consist of a way to assess the DIR uncertainty and a way to infer the dose warping error from that estimate.

Proposed methods to find the directional DIR uncertainty include iteratively making small modifications to the input images [56], performing registrations using slightly different algorithm configurations [57], and creating a set of test images with known deformations using a registration result and subsequently adding noise [58]. Proposed methods to then find the impact on the warped dose include using the covariance matrix of the transformations to blur the dose [56], creating a distribution of error maps that are used to warp the dose distribution [57], and comparing the warped dose to the result from the deformations used to create the image [58].

Advantages of these methods are that they include the effect of the direction of the registration variability and no new dose computation is needed. Their disadvantage is that the sensitivity of an algorithm to small changes in the images or its configuration is not necessarily linked to its registration uncertainty. The usability of the algorithm-generated deformations will depend on the algorithm used, and the interpretation of the result is not straightforward.

## 3 Phantoms for commissioning and quality assurance of DIR

Physical phantoms are recommended for end-to-end testing of DIR –especially for multi-modal registrations, with the caveats that they lack realistic anatomical features with sufficient image information to test the DIR and that they either lack realistic deformations or a full ground truth [59], [60]. For commissioning rigid registration, (simple) physical phantoms may be sufficient. For commissioning of DIR, especially for (intra-fraction) dose warping applications, more complex deformable phantoms are needed. Additionally, there is a need for anatomy-specific phantoms as imaging and deformations differ by anatomical region. An important step in this direction can be achieved by openly sharing data from physical phantoms, but there are practical limitations such as accessibility to the same phantoms, financial implications, multi-modal use, and the fact that some phantoms are in-house developed.

For commissioning of dose warping methodology, the ideal scenario is to directly measure deformed dose distributions to use as a ground truth using deformable 3D-dosimeters [61], [62], [63]. However, this laborious measurement process is neither applicable to emulate clinical scenarios nor readily accessible in routine clinics.

Virtual phantoms thus provide an important addition to physical phantoms. There is a dire need for a wide range of anatomies, image modalities, and types of deformations. Especially daily MR and multimodal images with (contours and) manually annotated landmarks are in high demand. In addition, biomechanical models can provide tests for complex and realistic anatomical deformations with a voxel-by-voxel benchmark. This can be useful for evaluating (intra-fraction) dose warping [11], [64]. Currently, commercial (clinical-grade) biomechanical models are lacking. The commercial software toolkit ImSimQA creates artificial deformations using piecewise polynomials that can be made to mimic clinically observed deformations for validation using locally acquired data [40].

Neylon *et al* propose a semi-automatic framework for the creation of patient-specific biomechanical models [65]. The GPU acceleration allows for interactive simulations. In this work, the user could rigidly deform the skeletal structures of a head & neck anatomy, whereafter the muscles and soft tissue deformations are governed by the modelled elastic interactions. The realistic known deformations can be used to test a registration algorithm for a specific patient’s anatomy. Neither physical nor digital phantoms are currently able to realistically model the mass changes required for inter-fraction dose warping validation.

We endorse a list of open-source dataset available in Supplement Material A in ‘MIRSIG position paper’ from the Australasian College of Physical Scientists and Engineers in Medicine (ACPSEM) [66], which is an evolving document available on the ACPSEM website^1^.

## 4 Recommendations

We endorse the recommendations by the AAPM Radiation Therapy Committee Task Group No. 132 [9], Barber *et al.* [67], and Paganelli *et al.* [68] and expand to recommend the following application-specific tools for commissioning and QA of (patient-specific) DIR.

*For contour propagation.* For (patient-specific) QA, use visual inspection of the registered image and contour(s). For commissioning, quantify the performance with the distance to agreement, comparing the registered contour to expert delineations.

**Fig. 4:**
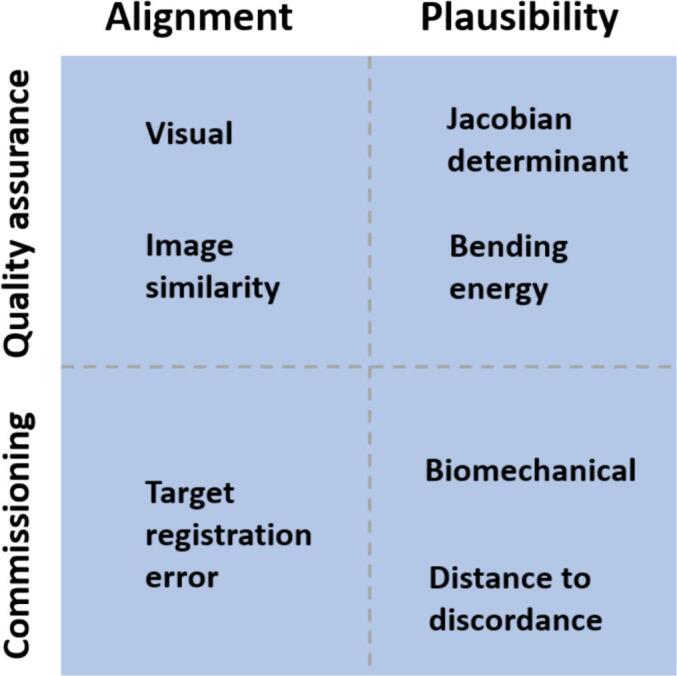
Recommendations on metrics for commissioning and quality assurance of DIR for warping quantitative information.

*For warping quantitative information*. Any evaluation should consist of *alignment metrics* (visual inspection, image similarity, target registration error) using registered images/contours/landmarks and *plausibility metrics* (Jacobian determinant, bending energy, biomechanical criteria, distance to discordance, inverse consistency, registration consistency) using the deformation vector field. Alignment metrics will flag up misalignments of contrast, while plausibility metrics will flag up implausible estimated deformations in low contrast areas. A combination of different tools using different inputs further reduces the impact of their respective limitations and insensitivities. See Fig. 4 for the overview.

**Fig. 5:**
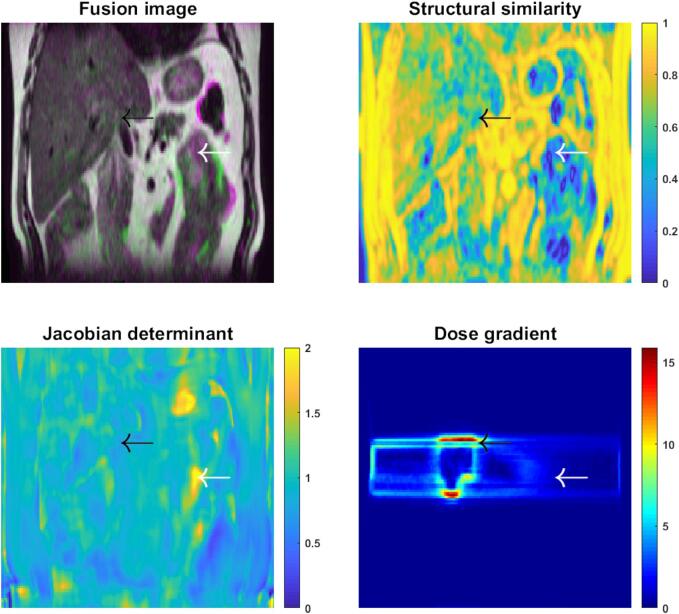
Example of quality assurance for dose warping on a liver anatomy showing a visual inspection using a fusion image; image similarity using structural similarity; transformation plausibility using the Jacobian determinant; and the dose gradient vector magnitude. Interesting areas are indicated with the black arrow (high dose gradient, low image similarity) and white arrow (low transformation plausibility, low image similarity).

In particular, *for QA of DIR for warping quantitative information,* we recommend at least visual inspection of image similarity and potentially the DVF for alignment. For plausibility, at least a voxel-wise Jacobian determinant and/or bending energy. For dose warping specifically, visualize the distance to dose difference or dose gradient. Registration errors in areas of high dose gradients are most relevant and harmful. See Fig. 5 for an example. *For commissioning of DIR for warping quantitative information,* we recommend the target registration error of a dense set of manually annotated anatomical landmarks for alignment, supplemented by the tissue-specific tensile and shear mechanical stress metrics for plausibility. If multiple images are available, the distance to discordance metric can be used. When evaluating the warping of Hounsfield units, we recommend the dosimetric comparison to a newly acquired CT. For dose warping, we recommend the use of virtual phantoms and some of the tools directly assessing the dose warping uncertainty [56], [57], [58], while taking their limitations into account.

We acknowledge that many of these tools as a minimum requirement are currently unavailable in many commercial software (see Supplementary Table S1), and encourage vendors to incorporate the recommended tools into their clinical solutions in the near future.

For interpretation, outputs in millimetres should be compared to the 2–3 mm thresholds set by the AAPM task group [9], to the voxel resolution of the images, to the segmentation uncertainty [69], and/or other clinically used (dose) thresholds. In these comparisons, it is important to consider the intended application as well as the complexity of the deformations and degree of movement, which depend on the anatomical region. In the prostate region, for example, one might expect the registration to be able to resolve most deformations, while in the abdomen one expects more challenges. Beyond general limits or tolerances, we recommend for alignment metrics to look at the relative improvement compared to values before (deformable) registration. For plausibility metrics, anatomy-specific tolerances and ranges should be considered.

The outputs from QA for DIR should be compared to the results in case no registration is performed. An imperfect registration might be more useful than no alignment at all.

Neural networks offer the possibility to use both image intensity and the deformation vector field to infer a registration error. They have shown promise as a useful independent contribution for this purpose, but currently require more validation as well as clear tests and descriptions of their applicability. Our recommendation is to use these methods with caution, if at all.

## 5 Discussion

A fundamental dilemma with evaluating registrations is that any automatic metric to evaluate the registration can also potentially be used to optimise the registration. If we want the best possible registration results, then we want to directly optimise the metrics that best indicate if the result is optimal- but then these metrics cannot be used for an unbiased evaluation of the registration result. Hence our recommendation of using an evaluation based on manual input for commissioning. Related, many DIR algorithms explicitly model transformations to be smooth, diffeomorphic, and/or incompressible. As with the evaluations using image similarity metrics that also drive the registration, evaluations with these metrics can still give useful information in these cases but should be used with caution. Note that our recommendation of evaluating both alignment and plausibility, is also related to how many registration algorithms work. These algorithms aim to optimize both a data fidelity term (determining *what goes where*) and a regularisation term (determining *how it goes there*). The former is evaluated using alignment metrics and the latter using plausibility metrics.

When applying DIR to dose warping and accumulation, the goal of the evaluation is less clear than for contour alignment. For the process of dose warping itself, many different views exist on what should and should not be done, and how. This subject is addressed in the ESTRO Physics Workshop review paper [14]. The difficulty with deforming dose is that it is often uncertain what exactly is the desired result. This is especially the case for inter-fraction cases where tissue might have (dis)appeared. In these cases, caution should be taken, and a better understanding of tissue changes is needed to really determine how to act [70], as in these cases image registration (and warping) is an ill posed problem [71]. Therefore, no benchmark or ground-truth exists in these cases, which makes the validation of dose warping challenging. On the other hand, we may be able to determine the most useful result for the intended application − e.g. if the application is to better estimate delivered dose for dose-outcome studies, then the methods of estimating the delivered dose that gives the strongest relationship with the outcomes is arguably the best. Note that if tissue is conserved, the algorithm may still estimate volume variations and the Jacobian determinant will help to interpret the resulted warped dose.

Recent ESTRO Physics Workshops on plan summation have discussed the potential benefit of using image registration in the process. The 2022 workshop on *re-irradiation: improving dose summation for plan optimisation and evaluation* resulted in a paper showing that a standardised process for dose summation based on image registration improved consistency in the accumulated dose between 24 participants [72]. The 2023 workshop on *methods to combine and sum external beam and brachytherapy dose distributions* recognized the important future role of DIR for dose combination, as it can provide a voxelwise addition. The workshop also indicated current limitations in DIR methods and the need for uncertainty and QA management. It is important to use the appropriate registration method and be aware of the remaining uncertainties.

When images contain artefacts, the registration is expected to fail locally. Image similarity and plausibility metrics should be able to flag these cases, such that action can be taken. In general, when images are unusable in certain regions due to artefacts, the registration should not be guided by image similarity there. An operator should choose to locally either use only the regularisation (imagine e.g. an artefact in the centre of the liver, it is expected that this moves similarly to the surrounding liver tissue) or to use additional information based on expert input, such as contours or points as guidance [73], [74], [75], [76], [47]. This can be used as a strategy to adapt an unsatisfactory registration result due to other reasons as well.

For many metrics discussed in this work the interpretation of their output values is not straightforward. This is particularly the case for the metrics recommended for evaluations regarding the warping of quantitative information. For alignment metrics, it is instructive to compare (distributions of) values to those obtained before registration. When the alignment has considerably improved, using plausible and consistent deformations in relevant areas, the result is sufficient for warping the quantitative information. Additionally, when comparing metric values between institutions (as well as registration algorithms, anatomies, cases), it is important that a metric is *universal* and generalizes over implementations. Work by Gooding *et al* found that there can be considerable differences between institutions and implementations when computing the same metric on the same data [18].

Recent developments in adaptive image-guided radiotherapy require application-specific tools for quality assurance and commissioning of image registration. While there is no single ideal evaluation tool, a combination of complimentary tools can provide an adequate assessment. A visual inspection is the foundation of any evaluation. Metrics based on manual input provide an independent evaluation when commissioning. Virtual phantoms are useful for commissioning of dose warping. When warping quantities of interest such as dose, many or all relevant voxels should be evaluated. For this, alignment metrics (e.g. image similarity, target registration error) should be combined with plausibility metrics (e.g. Jacobian determinant, biomechanical criteria, distance to discordance, registration consistency). For interpretation, alignment metrics should be compared to results before registration.

## Funding

LB, JM and CZ received funding from Elekta. MGJ reports speaker honoraria from Elekta, licencing agreement with Standard Imaging and institutional research agreements with Elekta and MIM.

JM is supported by a CRUK Centres Network Accelerator Award Grant (A21993) to the ART-NET consortium and by the Wellcome/EPSRC Centre for Interventional and Surgical Sciences (WEISS) (203145/Z/16/Z).

KB received funding from RaySearch Laboratories AB through a Co-Development and Collaboration Agreement and has a licensing agreement with RaySearch Laboratories AB.

LB and CZ received funding from the PPP Allowance made available by Health ∼ Holland, Top Sector Life Sciences & Health, to stimulate public–private partnerships and from the Dutch Research Council (NWO) through project no. 18495 (ADEQUATE).

## CRediT authorship contribution statement

**Lando S. Bosma:** Conceptualization, Investigation, Methodology, Visualization, Writing – original draft. **Mohammad Hussein:** Conceptualization, Project administration, Resources, Writing – review & editing. **Michael G. Jameson:** Conceptualization, Project administration, Resources, Writing – review & editing. **Soban Asghar:** Conceptualization, Writing – review & editing. **Kristy K. Brock:** Conceptualization, Writing – review & editing. **Jamie R. McClelland:** Conceptualization, Methodology, Writing – review & editing. **Sara Poeta:** Conceptualization, Writing – review & editing. **Johnson Yuen:** Conceptualization, Writing – review & editing. **Cornel Zachiu:** Conceptualization, Methodology, Writing – review & editing. **Adam U. Yeo:** Conceptualization, Supervision, Writing – review & editing.

## Declaration of competing interest

The authors declare that they have no known competing financial interests or personal relationships that could have appeared to influence the work reported in this paper.

## Footnotes

1 https://www.acpsem.org.au/About-the-College/Special-Interest-Groups/MIRSIG.
