# Supplementary material for: Tools and recommendations for commissioning and quality assurance of deformable image registration in radiotherapy
Source: Phys Imaging Radiat Oncol. 2024 Sep 14;32:100647. doi: 10.1016/j.phro.2024.100647 (PMC11424976; doi:10.1016/j.phro.2024.100647)
Supplement: Supplementary Data 1 [file mmc1.pdf]

Supplementary Material

Table S1. Overview of metrics used in different commercial software with at least 5% international use as found by Yuen et al [4]. Information collected from the literature and conversations at ESTRO 2022 in Copenhagen. The metrics are visual inspection of the registered image, visual inspection of the deformation vector field (DVF), the distance to agreement (DTA), the Dice similarity metric (Dice), the target registration error (TRE), image similarity (ImSim), consistency metrics (Consistency), the Jacobian determinant (JacDet), bending energy (BendEn), biomechanical criteria (Biomech), and dose-based metrics.

Software	Visual image	Visual DVF	DTA/ Dice	TRE	ImSim	Consistency	JacDet	BendEn	Biomech	Dose based
MIM[1]	✓	✓	✓		✓[2]		✓			
Velocity[3]	✓	✓	✓	✓			✓			
Mirada RTx[4]	✓	✓								
Raystation TPS [5]	✓	✓	✓	✓	✓		✓			
Pinnacle3 TPS[6]		✓								
Eclipse TPS	✓	✓	✓	✓			✓			

---

[1] https://www.mimsoftware.com.cn/manager/templates/mimsoftware/resources/abstracts/ASTRO%2013%20POPI%20Reg%20Reveal.pdf and https://doi.org/10.1016/j.ijrobp.2013.06.1903.
[2] Global value only
[3] https://varian.widen.net/view/pdf/dizhixzecz/VelocityClinicalPerspectives_RAD10438_February2018.pdf?u=bmxzem from https://www.varian.com/products/interventional-solutions/velocity
[4] https://www.mirada-medical.com/miradartx
[5] https://www.raysearchlabs.com/49e915/siteassets/about-overview/media-center/wp-re-ev-n-pdfs/white-papers/deformable-registration-in-raystation2.pdf
[6] https://www.usa.philips.com/healthcare/solutions/radiation-oncology/radiation-treatment-planning
